# Flu vaccination coverage in healthcare workers during a 3-year period in the context of the pandemic: Giuditta Scardina

**DOI:** 10.1093/eurpub/ckac129.597

**Published:** 2022-10-25

**Authors:** G Scardina, L Ceccarelli, V Casigliani, S Mazzilli, M Padovan, A Petillo, L Tavoschi, R Foddis, G Privitera, A Baggiani

**Affiliations:** 1 Translational Research in Medicine, University of Pisa, Pisa, Italy; 2 Scuola Normale di Pisa, Pisa, Italy; 3 Occupational and Preventive Medicine, University of Pisa, Pisa, Italy

## Abstract

**Background:**

Vaccination of healthcare workers (HCWs) against seasonal influenza is considered the most effective way to protect HCWs and maintain essential healthcare services during influenza epidemics. With the present study we aimed to evaluate the efficacy of measures implemented during the three flu campaigns of 2018/19, 2019/20 and 2020/21 in a university hospital in Pisa, Italy, through the assessment of vaccination coverage (VC) in HCWs and to assess attitudes toward flu vaccination.

**Methods:**

Flu VC was stratified according to sex, age, job and vaccination status for each season and the association between each variable and vaccination status was explored. In 2020, a survey collecting data on knowledge and attitudes on flu vaccination was distributed.

**Results:**

Starting from the 2018/19 campaign, an increasing flu VC rate was registered: contained in 2019/20 (from 11.6% to 14.3%, Δ%=23.1) and significant (VC = 39.6%, Δ%=177.6) in 2020/21 as compared with the previous year. Physicians were the professionals most willing to get vaccinated during all seasons. Considering age the situation changed greatly over the study period, with VC rising in 2020/21 in those age groups marked by low VC in previous years (<30 and 41-50 years old, Δ%=293). Having been vaccinated in the previous year represented the most important variable to predict likelihood of accepting flu jab. However, while previously vaccinated HCWs were 13 times more likely to get the flu jab in 2019/20 compared with the others; in 2020/21 they were only 3 times. Only half of HCWS considered themselves at higher risk of contracting influenza compared to the general population, while 71% totally agreed that receiving the flu jab in 2020/21 was more important than the previous years due to COVID.

**Conclusions:**

A significant increase in VC was observed in 2020/21, especially among those sub-groups with consistently lower uptake in previous years, due to the COVID pandemic that positively influenced vaccination uptake.

**Key messages:**

• This study evaluates the impact of subsequent flu vaccination campaigns implemented in a large university hospital in Italy through the assessment of flu VC among HCWs.

• A significant increase in flu VC among HCWs was observed in 2020/21, especially in those categories characterized by lower VC rates in the previous years, more likely due to the COVID-19 pandemic.

